# Recording the maxillomandibular relationship with the Aqualizer system prior to occlusal splint therapy for treating temporomandibular disorders: a randomized controlled trial

**DOI:** 10.1038/s41598-023-49911-7

**Published:** 2023-12-18

**Authors:** Karin Christine Huth, Alexandra Bex, Maximilian Kollmuss, Sabina Noreen Wuersching

**Affiliations:** Department of Conservative Dentistry and Periodontology, LMU University Hospital, LMU Munich, Goethestrasse 70, 80336 Munich, Germany

**Keywords:** Oral diseases, Chronic pain

## Abstract

Temporomandibular disorders (TMD) present a public health issue and are one of the most common musculoskeletal conditions causing chronic pain. This study compares the outcomes of occlusal splint therapy in patients with TMD following two different maxillomandibular relationship (MMR) registration techniques. 40 TMD patients were randomly allocated to MMR registration with the Aqualizer system (AQU) or with chin point guidance (CPG) prior to fabricating occlusal splints. TMD symptoms, subjective pain intensity, and quality of life (QoL) were recorded at baseline and after 3 and 6 months. The treatment led to an overall reduction of TMD symptoms in both groups (Conover test, p < 0.00001). TMJ sounds, TMJ pain with palpation and muscle pain with palpation subsided regardless of the type of MMR registration method used (Cohen’s d > 0.8). AQU-based occlusal splints led to a better improvement of TMJ pain with maximum opening compared to CPG-based occlusal splints (Cohen’s d = 0.9; CPG d = 0.13). In both groups, occlusal splint treatment had little to no effect on correcting lateral mandible deviation or improving restricted jaw opening. After 6 months occlusal splints in both groups had a large effect on improving subjective pain intensity (Cohen’s d > 0.8), however, patients reported a higher QoL in the AQU group compared to the CPG group (Mann–Whitney-*U*-test, p < 0.05). The results of this study support the premise that occlusal splints are effective in relieving pain-related TMD symptoms. The Aqualizer can be considered for determining MMR in cases, where guided registration techniques are not possible.

Trial registration: DRKS00031998.

## Introduction

Temporomandibular disorders (TMDs) are the most common musculoskeletal conditions in the maxillofacial region involving the temporomandibular joint (TMJ) complex and the surrounding muscular and osseous components. According to the U.S. National Institute of Dental and Craniofacial Research, TMDs affect approximately 5 to 12% of the population and have their highest prevalence among younger women between 20 and 40 years of age^1,2^. The most common symptoms of TMDs are chronic pain in the area of the TMJ, regional myofascial pain, and functional limitations of the jaw movement^3^. Patients suffering from disk displacement disorders may report clicking, popping, or grating sounds in the TMJ or show lateral mandible deviation on opening. TMJ pain radiating to the surrounding structures can lead to chronic headaches, earaches or tenderness in the neck or back muscles, which may present a psychological burden and impact the quality of life of the affected individuals^4^.

Common conservative treatment methods for relieving TMJ pain include physical therapy, relaxation exercises and pharmacological therapy^5^. However, many patients, particularly those suffering from pain-related TMD, seek consultation with dentists, whose first line of treatment is typically the use of occlusal splints. Although the evidence for the efficacy of oral splints remains moderate to low, occlusal splints have been shown to alleviate TMD-related pain and improve functional limitations of the jaw^6–8^. The main purpose of occlusal splints is to achieve neuromuscular conditioning by disengaging the occlusion and bringing the condyles into a position that supports relaxation of the masticatory muscles, enables the repositioning of the articular disk and balances proprioception of the periodontal ligament^9,10^. Therefore, recording the maxillomandibular relationship (MMR) is an important step prior to manufacturing occlusal splints for TMD patients. The centric relation (CR) position is a typical MMR position that has been repeatedly suggested for TMD patients^11–13^. CR is considered a physiological position of the jaw, where the condyles articulate with the thinnest avascular intermediate zone of their respective disks^14^. Despite the concept of CR having been studied for almost 100 years, the clinical relevance of positioning the condyles in CR in TMD patients has been controversially discussed^15,16^.

The most popular traditional MMR registration techniques are bilateral manipulation and chin point guidance, which are both considered operator-determined methods, because the mandible is guided into the desired position by the dentist^17–20^. A common variation of these techniques is the insertion of an anterior jig, which prevents the posterior teeth from occluding and, in doing so, enables neuromuscular deprogramming by eliminating proprioceptive stimuli from the periodontal ligament and the surrounding musculature^21^. Although chin point guidance is considered to provide the most reliable results, the problem with this technique is that it is difficult to master and the registration accuracy is therefore greatly dependent on the dentist’s experience^22,23^. Furthermore, excessive forces applied unintentionally during mandibular guidance can easily place the condyles in an unfavorable position causing further discomfort^18^. Therefore, patient-guided MMR registration methods, such as gothic arch tracing, leaf gauges and swallowing technique, have been advocated for being at lower risk of forcing the mandible into a non-physiological position and allowing the patient’s anatomical components to establish the desired MMR position more naturally.

Recently, hydrostatic oral splints have been proposed as novel patient-guided method for recording the MMR. The Aqualizer is a self-adjusting oral device consisting of two water-filled fluid pads and a connecting tube, which form a waterbed system that distributes the fluid evenly when pressure is applied. According to the manufacturer, this device acts as a deprogrammer and balances occlusal forces, thereby facilitating repositioning of the mandible and the surrounding muscles in their most comfortable position. Previous studies have shown that short-term treatment with the Aqualizer can relieve TMD-related pain, improve functional limitations and lead to an improved sleep quality^24–27^. Therefore, the Aqualizer may also be a promising in-office device for bite registration prior to manufacturing oral splints, assuming that it enables the patient’s stomatognathic system to naturally balance itself without guidance by the dentist. This present study aims to compare the effect of occlusal splint treatment following MMR registration with the Aqualizer and chin point guidance in TMD patients. To compare the effect of either MMR registration technique on the outcomes of occlusal splint treatment, additional TMD treatment methods, such as physical therapy, were omitted. The primary aim is to evaluate whether there is a significant difference in treatment outcomes, that is, symptom alleviation and reduction of subjective pain intensity, between these two MMR registration methods. The second aim is to examine the patients’ perception of improvement in quality of life (QoL), patient satisfaction with the entire treatment, and the occlusal fit of the splints following the different registration techniques.

## Results

**Figure 1:**
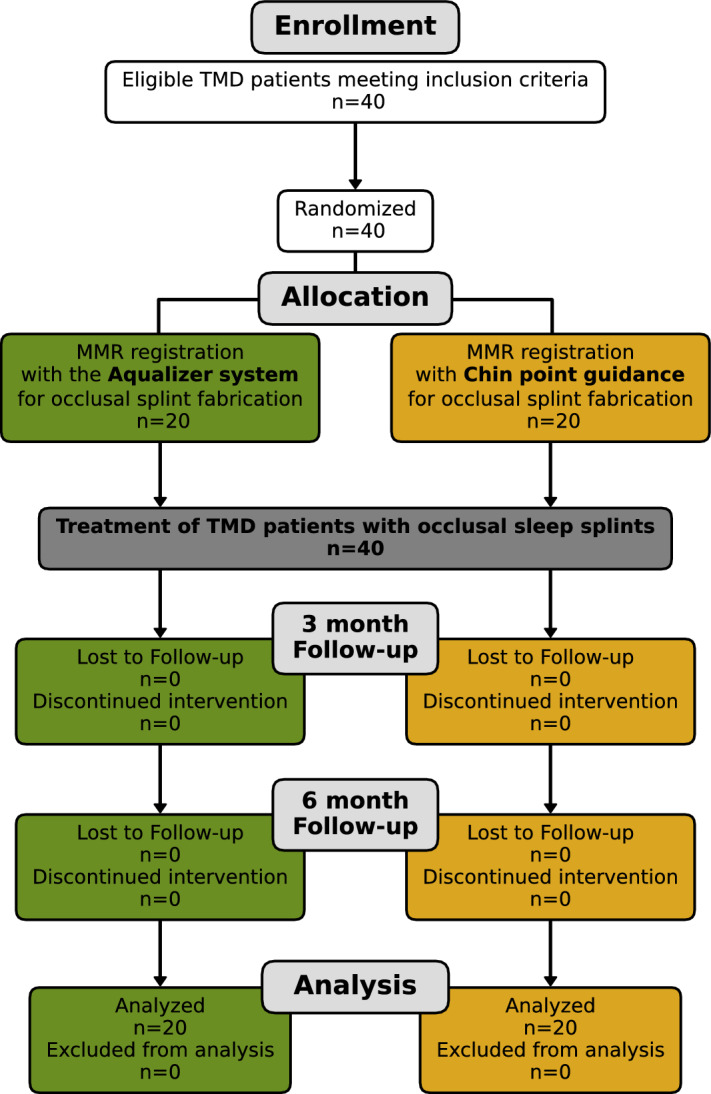
Flowchart of trial phases according to CONSORT guidelines.

The CONSORT Flowchart showing the trial design is displayed in Fig. 1. Demographic data of the study populations were similar in both groups and showed no significant difference for any of the variables (Table 1).

**Table 1:** Demographic data of study population in each group. No significant differences between AQU and CPG groups were observed for any of the variables (Mann–Whitney *U* test, Fisher’s exact test where applicable).

Variable	AQU Group (n = 20)	CPG Group (n = 20)	p-value
Average age in years (mean ± SD)	25.6 ± 3.5	29.4 ± 12.9	0.94
Gender (male/female)	5/15	6/14	1.00
Average number of self-reported TMD-related symptoms (I)–(IV) (mean ± SD)	2.25 ± 0.79	2.70 ± 0.92	0.06
(I) Headaches and/or neck pain	10 (50%)	13 (65%)	0.52
(II) Cracking/grinding in the temporomandibular joint	13 (65%)	15 (75%)	0.73
(III) Pain in the area of the temporomandibular joint and/or the surrounding muscles	18 (90%)	20 (100%)	0.49
(IV) Functional restrictions of the jaw movement	4 (20%)	6 (30%)	0.72

### Primary outcomes

#### Reduction of TMD symptoms

**Figure 2:**
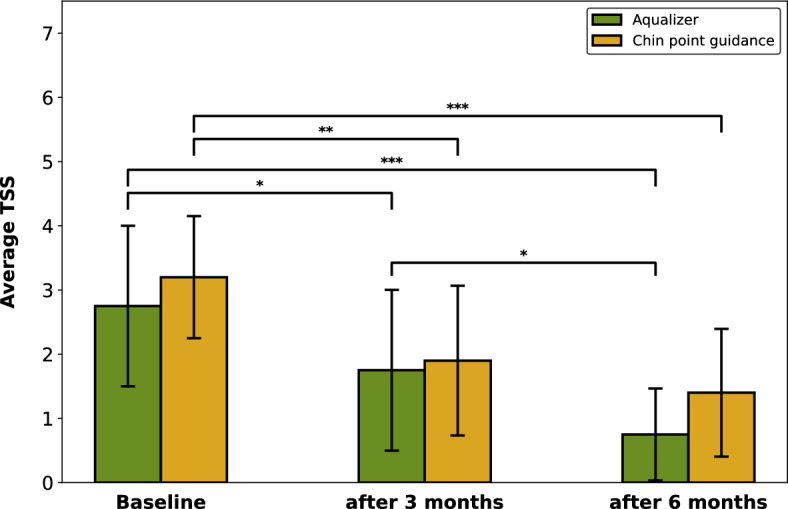
Average TMD symptom score (TSS) at baseline and after 3 and 6 months of occlusal splint therapy. P-values determined by the Friedman test and Conover post hoc test with a Holm–Bonferroni correction (*p < 0.05; **p < 0.0001; ***p < 0.00001).

The presence of TMD symptoms #1–6 at baseline and after 3 and 6 months of splint treatment are shown in Table 2. The average total TMD symptom score (TSS, sum of symptoms #1–6 for each patient) is visualized in Fig. 2. In both groups, occlusal splint therapy led to a significant reduction of the TSS after 3 and 6 months (Conover post hoc test, p < 0.00001). In the Aqualizer (AQU) group, there was an additional, significant TSS reduction between 3 and 6 months. Pairwise comparisons of the TSS in the AQU and chin point guidance (CPG) group at each observation point indicated a significant difference after 6 months (p < 0.05) in favor of the AQU-based occlusal splints.

**Table 2:** Primary and secondary outcomes after occlusal splint treatment.

	AQU group			CPG group		
	Baseline	3 months	6 months	Baseline	3 months	6 months
#1 lateral mandible deviation on opening	12	11	8	12	12	12
#2 limited jaw opening	1	1	1	1	1	1
#3 TMJ sounds	13	6	5	15	8	8
#4 TMJ pain with maximum opening	6	5	0	4	4	3
#5 pain with TMJ palpation	8	2	0	12	2	0
#6 pain with muscle palpation	15	10	1	20	11	4
Occlusal adjustments	18	3	0	17	5	3

**Figure 3:**
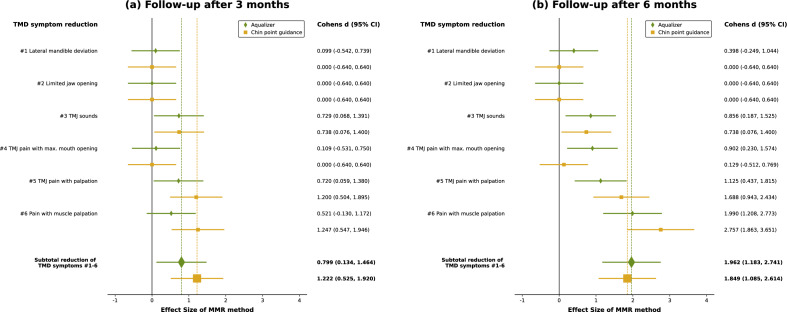
Forest plot for visualizing the effect sizes of the MMR registration method on the reduction of TMD symptoms #1–6 after Aqualizer- or Chin point guidance-based occlusal splint treatment for (**a**) 3 months and (**b**) 6 months. Effect sizes were estimated by computation of Cohen’s d and 95% confidence intervals (CI).

For the purpose of comparing the magnitude of the TSS reduction achieved by AQU- or CPG-based splint therapy, the effect sizes for each symptom were determined at the 3 months- and 6 months follow-up and visualized in a forest plot (Fig. 3). At the 3-month recall visit, both AQU- and CPG-based occlusal splints seemed to have had a large effect on reducing TMJ sounds (#3) and pain with TMJ palpation (#5). As far as lateral mandible deviation on opening (#1), limited jaw opening (#2), and TMJ pain with maximum opening (#4) are concerned, the confidence intervals in both groups crossed the line of no effect (x = 0), implying that neither registration method had a significant effect on the reduction of these symptoms after 3 months. This also applies to the AQU group regarding pain with muscle palpation (#6), whereas in the CPG group, occlusal splint treatment seemed to have had a significant effect on alleviating this symptom. The effect estimation implies that after a treatment period of 6 months, both registration methods had a large effect on reducing TMJ sounds (#3), pain with TMJ palpation (#5) and pain with muscle palpation (#6). Additionally, AQU-based occlusal splints had a significant effect on TMJ pain with maximum opening (#4). Both occlusal splints still had little to no effect on lateral mandible deviation on opening (#1) and limited jaw opening (#2) after 6 months. The total effect of occlusal splint therapy on the reduction of TMD symptoms was rated as large for both registration methods. Although the effect size for the subtotal reduction of all symptoms was larger initially in the CPG group, the overall effect after 6 months was similar in both study groups.

#### Subjective pain intensity

**Figure 4:**
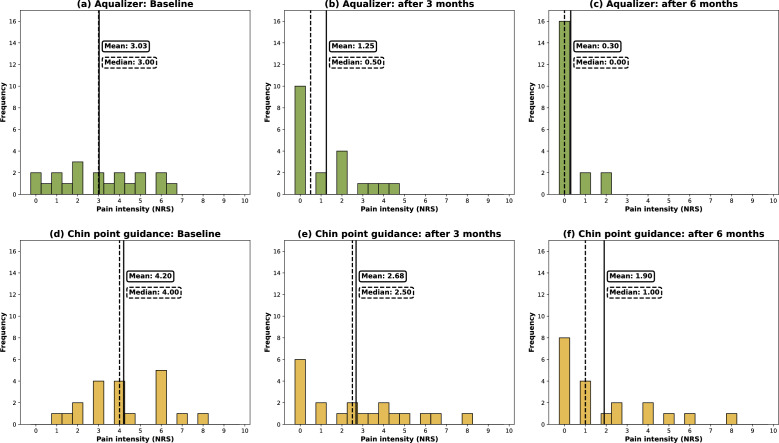
Histograms showing the frequency of pain intensity scores 1–10 at baseline and after 3 and 6 months of occlusal splint therapy following MMR registration with the Aqualizer system (**a**–**c**) or chin point guidance (**d**–**f**). Vertical lines represent mean (solid line) and median (dashed line) pain intensity.

Changes in subjective pain intensity were recorded using a numerical rating scale (NRS). The frequency of the NRS scores at each visit are displayed in histograms (Fig. 4). Mean and median pain intensity declined after 3 and 6 months of occlusal splint treatment in both groups. The effect estimation implies that 3 months of splint treatment had a large effect on the reduction of subjective pain intensity in both groups (AQU: Cohen’s d = 0.98, CI: 0.30, 1.66; CPG: Cohen’s d = 0.69, CI: 0.03, 1.34). This effect further increased after a treatment period of 6 months in both groups (AQU: Cohen’s d = 1.79, CI: 1.03, 2.54; CPG: Cohen’s d = 1.07; CI: 0.38, 1.75).

### Secondary outcomes

#### Quality of Life

**Figure 5:**
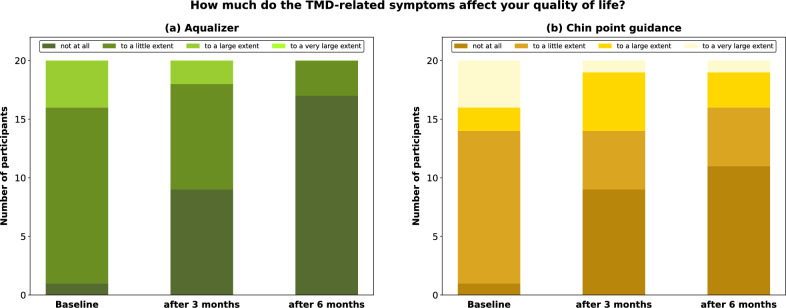
Patient perception on the impact of occlusal splint therapy on quality of life.

The patient perception on the impact of occlusal splint therapy on Quality of life (QoL) is displayed in Fig. 5. In both study groups, an improvement in QoL was observed after 3 and 6 months of occlusal splint therapy (Friedman test, p < 0.0001). In comparison to the baseline data, the average QoL in the AQU group improved significantly after 3 (p < 0.05) and 6 months (p < 0.0001), whereas a significant improvement in QoL in the CPG group was only observed after 6 months (p < 0.05). Furthermore, the average QoL was significantly higher in the AQU group compared to the CPG group after 6 months (p < 0.05).

#### Patient satisfaction

**Figure 6:**
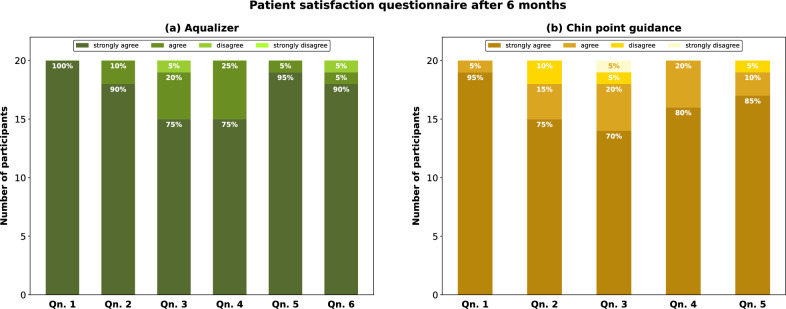
Results of the patient satisfaction questionnaire completed at the 6 months recall visit. Qn. 1 General satisfaction: I am satisfied with the entire treatment I received from start to finish. Qn. 2 Expectations: My expectations to the treatment were fulfilled. Qn. 3 Alleviation: The treatment helped alleviate my initial discomforts. Qn. 4 Wearing comfort: I did not encounter any problems while wearing the occlusal splint. Qn. 5 Recommendation: I recommend this type of treatment. Qn. 6 Comfort: Bite registration with the Aqualizer system was not uncomfortable (only AQU Group).

The results from the patient satisfaction questionnaire (PSQ) are displayed in Fig. 6. In both groups, none of the participants found the occlusal splint uncomfortable to wear and most patients in the AQU group also perceived the MMR registration with the Aqualizer as comfortable. Two participants in each group reported having a dry mouth while wearing the occlusal splint. One patient in the AQU group and two patients in the CPG group did not feel that their initial discomforts were sufficiently alleviated by the treatment. Nonetheless, all patients were satisfied with the entire treatment from start to finish.

#### Occlusal adjustments

Occlusal adjustments were necessary in 18 AQU patients and 17 CPG patients upon insertion of the occlusal splint (Table 2). Further adjustments after 3 months of wearing the splint were necessary in both groups. At the 6-month recall visit, no occlusal interferences appeared in the AQU group, whereas three occlusal splints in the CPG group required further adjustments.

## Discussion

This study examined whether the Aqualizer is a suitable patient-guided method for determining MMR prior to occlusal splint treatment. Occlusal splints are a frequently used non-invasive treatment approach for patients with TMD. However, there is conflicting evidence for the efficacy of occlusal splints, with some studies finding effectiveness in pain reduction and others finding no symptom alleviation regarding pain, clicking of the TMJ, and functional restrictions^8,27,28^. Moreover, it has been questioned whether CR is a prerequisite for occlusal splints. The definition of CR itself is controversial, as multiple definitions have evolved over time, ranging from the most posterior position to a superior position and the anterior–superior position in which the condyles articulate against the posterior slopes of the articular eminences^10,29^. There still seems to be little scientific evidence to support any proposition as to where the exact position of the condyle should be in relation to the fossa^16^. Thus, there is also little consensus on the ideal method for recording the MMR. Operator-guided methods such as CPG or bimanual manipulation are established techniques and are often favored by clinicians. However, these methods may not always be suitable, especially for inexperienced practitioners, or in cases, where excessive forces are needed to guide the mandible into the desired MMR position, such as in patients presenting with acute TMJ pain or patients with masticatory muscle hyperactivity. In these cases, self-adjusting and/or patient-guided techniques may be useful for achieving an unstrained and natural position of the jaw. The principle of the Aqualizer is based on Pascal’s law, which states that an increase in pressure at any point in an enclosed fluid leads to an equal increase at every other point in the container. Under this premise, we hypothesized that the Aqualizer may be a useful tool for recording MMR prior to occlusal splint fabrication. Since the Aqualizer is a patient-guided MMR registration method, instructing the patient about the procedure is crucial to avoid unintentional lateral or sagittal misplacement of the mandible. Therefore, we recommend constant supervision by the dentist during MMR registration with the Aqualizer. It is important to note, that the Aqualizer leaves the position of the mandible up to the patient, which may not be the CR position as, at least in theory, achieved with guided registration methods. The only reference method to confirm the true position of the condyles in each patient is magnetic resonance imaging (MRI) of the TMJ, which we did not use in our study. However, our results suggest that MMR in CR may not be as essential as previously thought to achieve successful results in patients with TMD, as long as the MMR registration method brings the mandible in a comfortable position which is sufficient for fabricating the occlusal splint.

For this study, we focused primarily on patients reporting pain-related TMD symptoms correspondent to the twelve common TMD conditions described by the DC/TMD: arthralgia, myalgia, local myalgia, myofascial pain, myofascial pain with referral, disk displacement disorders, degenerative joint disease, subluxation, and headache attributed to TMD^4^. Furthermore, our study population represents typical TMD prevalence regarding age and gender distribution, as TMDs are reportedly most common in those 20 to 40 years of age and are more prevalent in women than in men^2^. Contrary to the studies mentioned above, our results support the premise that treatment with occlusal splints may be effective in relieving TMD symptoms, as we observed an overall reduction of the TSS in both our study groups. If we break down the effect the treatment had on each symptom, it is noticeable that after 6 months the estimated effect sizes for pain-related symptoms, such as TMJ pain with palpation and pain with muscle palpation, were greater in the CPG group compared to the AQU group. This may be attributed to the fact that there was a slightly skewed distribution of patients reporting pain-related symptoms, with the frequency of these symptoms leaning to the CPG group. Given that the presence of all symptoms was assessed dichotomously and that treatment with occlusal splints was effective in reducing a certain symptom, the mean difference used for computing the effect sizes becomes larger in those cases, where there were more subjects positive for that symptom at baseline. Hence, the effect size of occlusal splint treatment was larger in the CPG group regarding TMJ pain with palpation and pain with muscle palpation. However, the analysis of the overall subjective pain intensity, which was assessed quantitatively with a continuous scale (NRS), indicates that mean pain intensity was lowest in the AQU group after 6 months. As a result of the skewness regarding pain-related symptoms, the mean pain intensity was higher in the CGP group to begin with as well. In fact, there were two patients in the CPG group reporting a high NRS score (6 and 8) at baseline that stagnated throughout the entire treatment. The main cause of discomfort in one of these patients was TMJ pain with maximum opening and restricted jaw opening, which did not improve by means of occlusal splint treatment. The other patient’s primary discomforts were also signs of disk displacement disorders, that is, TMJ pain with maximum opening and clicking sounds in the area of the TMJ. For these patients, occlusal splint treatment was not successful in alleviating their main symptom, which implies that further diagnostics and treatment methods may be needed for such patients. For example, counselling therapy and therapeutic exercises have been shown to be effective in patients with disk displacement disorders and therefore should be considered as an additional treatment option^30^. Nonetheless, our results suggest that the treatment in both groups showed a comparable success rate in the reduction of subjective pain intensity, considering that the proportion, by which the median pain intensity was reduced, was similar in both groups (median difference in both groups 3.0).

To ensure similar wearing times among all study participants, the occlusal splints were mainly used as sleep splints in this study. Since occlusal splints with anterior tooth coverage usually impair articulatory phonetics, the average wearing time during the day would have severely varied depending on the participants’ profession. There are indications that the treatment period of occlusal splint therapy may be an important parameter for treating TMD. This is based on the observation that the effect sizes for the reduction of symptoms increased between 3 and 6 months in both groups. In fact, this increase in effectiveness led to a significant difference between the total number of symptoms (TSS) at 3 and 6 months in the AQU group. Furthermore, AQU-based splint treatment had no significant effect on relieving TMJ pain with maximum opening and pain with muscle palpation after 3 months, whereas after 6 months, both symptoms seemed to have improved significantly in nearly all patients. This implies that the occlusal splints based on the condyle-fossa relation provided by the Aqualizer exhibited their therapeutic effect later than splints fabricated on the basis of CPG. After 6 months of treatment, TMJ pain with palpation and muscle pain with palpation seemed to have subsided sufficiently regardless of the registration method. While TMJ pain with maximum opening improved for all patients in the AQU group, only one patient in the CPG group was free of this symptom at the end of the treatment. Interestingly, the presence of TMJ sounds improved after 3 months in both groups, but there was no further reduction of this symptom after another 3 months of treatment. This is also in agreement with a previous 6-months clinical trial, where joint sounds subsided in approximately 30% of the patients receiving occlusal splint treatment after a treatment time of 3 months, but no further reduction was observed after 6 months^31^. It has been suggested that TMJ sounds may decrease during occlusal splint treatment due to the increased joint space which allows a smoother condylar translation beyond disk surface irregularities^32,33^. Irrespective of the MMR registration method used, occlusal splint treatment seemed to have had little to no effect on correcting lateral mandible deviation on opening and improving restricted jaw opening. Perhaps symptoms indicative of disk displacement disorders or further joint diseases require a longer treatment period to reshape the disk-joint assembly into a physiologic position. It has been previously shown that the duration of occlusal splint treatment for improving restricted mouth opening may range from 6 to 24 months to restore a normal maximal interincisal distance^34^. Furthermore, it should be mentioned that the number of patients exhibiting these symptoms in our study are not representative for drawing conclusions about the effectiveness of splint treatment on improving restricted jaw opening.

The secondary outcomes of this study were to assess the patients’ perception of their QoL before, during and after the treatment, the patients’ general satisfaction with the treatment and the accuracy of the occlusal fit. The occlusal splint treatment led to an improved QoL in most participants, but QoL in the AQU group was significantly higher compared to the CPG group. After 6 months of treatment, there were more participants in the AQU group than in the CPG group reporting that their TMD-related symptoms did not affect their QoL at all. Similar results were also obtained from the PSQ. In both groups, all patients were generally satisfied with the entire treatment and found the occlusal splint comfortable to wear, implying that occlusal splints are a treatment method well-received by most patients. In both groups, the occlusal splints showed an overall good occlusal fit. The occlusal fit, judged by the number of interfering contacts, can be viewed as an indirect measure of the accuracy of the registration technique or the level of comfort of the MMR. Upon insertion, most patients required occlusal adjustments in both groups, however, fewer patients in the AQU group needed further adjustments at the follow-up visits. The fact that occlusal interferences were removed presents a limitation for purely comparing the success of occlusal splint treatment in the CPG and AQU group. Nonetheless, interfering contacts are considered a potential co-factor for causing or aggravating TMDs in susceptible patients, which is why we chose to eliminate all potential sources of further discomfort^35,36^. When drawing comparisons between MMR registration with the Aqualizer and CPG, one drawback of the Aqualizer that should be mentioned are the extra costs, which range from 20 to 30 US$ or 20 to 25€ depending on the country and the distributer.

As far as the choice of splint material is concerned, there are conflicting opinions as to which type of material is the most suitable for occlusal splints. Soft splints are advocated by some researchers because they were found to be effective in alleviating myogenous TMD symptoms^37,38^. However, other studies found that hard splints were more retentive than soft splints and more effective in reducing bite forces and treating both myogenous and arthrogenous TMD symptoms^28,39^. Among the several manufacturing methods for occlusal splints, subtractive and additive CAD/CAM techniques are nowadays the method of choice for most clinicians and dental technicians. This development is not only attributed to the increasing demand for digital workflows but is also due to the fact that these techniques have shown several advantages in terms of throughput, complexity, precision and customization compared to traditional manufacturing methods such as injection molding or vacuum-forming^40,41^. Furthermore, occlusal splints manufactured with additive and subtractive CAD/CAM methods have been shown to exhibit superior surface properties, making them less susceptible to bacterial adhesion and plaque accumulation^42^. In this study, the participants received a hard thermoplastic PMMA splint which becomes pliable upon exposure to higher temperatures, for example when placed in warm water. The patient can benefit from this feature by a facilitated insertion and an increased wearing comfort since the thermoplastic properties help maintain a precise internal fit over the entire treatment period. A comfortable and tension-free fit of occlusal appliances is crucial especially for TMD patients because pressure applied to the dental arch by a too tight fit can aggravate facial tension and headaches.

In general, the findings of this study must be interpreted in the light of several limitations. When studying TMD treatment strategies, it should be noted, that further conservative methods, such as home exercises and physical therapy, can be just as effective as occlusal splints in terms of symptom alleviation in the acute phase, possibly making occlusal splint treatment redundant^6^. Therefore, the significance of our results are limited by the fact that our study design is lacking a control group for further comparison. Perhaps the outcomes of our study would have been more apparent if there had been a third study group including TMD patients, who were only instructed to perform home exercises or being treated with physical therapy. Moreover, the TMD symptoms among the participants may have been too diverse for generalizing the outcomes of our study. Recruiting patients with the same set of symptoms is challenging due to the heterogeneous nature of TMD, making it more difficult to study the treatment effects based on the TMD categorization.

## Conclusions

Due to the large variation in diagnostic criteria and splint types found in clinical studies, there is a lack of standardized treatment regimens, which is one of the reasons for the low evidence of occlusal splint therapy in TMD patients. The results of our study indicate that occlusal splint treatment in general can alleviate pain-related TMD symptoms and lead to an improvement in QoL. Based on our results, the symptoms TMJ sounds, TMJ pain with palpation and muscle pain with palpation seem to subside over time regardless of the type of MMR registration method used. AQU-based occlusal splints led to a greater alleviation of TMJ pain with maximum opening than CPG-based occlusal splints. In neither group, the occlusal splint treatment was able to correct lateral mandible deviation or improve restricted jaw opening. The treatment of some TMD symptoms seemed to depend on the duration of occlusal splint therapy. In conclusion, the Aqualizer seems to be a suitable tool for recording MMR in TMD patients, however, the effect of AQU-based splints did not exceed that of CPG-based splints in this study. Therefore, the Aqualizer can be considered an alternative to CPG in cases, where operator-guided registration techniques are not possible.

## Materials and methods

### Trial design and ethical clearance

The present study was a prospective, randomized controlled clinical trial and adhered to the Consolidated Standards of Reporting Trials (CONSORT 2010) statement. Ethical approval was obtained from the Ethics Committee of the Medical Faculty of the University of Munich (registration no. 20-681). Informed written consent was obtained from all patients participating in this study. The investigation was conducted as a single-center study at the Department of Conservative Dentistry and Periodontology of the University Hospital, LMU Munich from October 2020 to October 2021.

### Participants

Patients entering the outpatient unit of the department were screened for the following eligibility criteria: healthy adults over 18 years of age reporting typical TMD symptoms such as (I) chronic headaches and/or neck pain, (II) cracking/grinding sounds in the TMJ, (III) recurrent pain in the area of the TMJ and/or the surrounding muscles, (IV) functional limitations of the jaw movement. Patients who met at least one of the criteria (II)–(IV) were enrolled into the study. Those solely reporting (I) chronic headaches and/or neck pain were not included in this study due to this criterion being least unique to TMD. Exclusion criteria were patients under 18 years of age, unwillingness to participate in the study, partial edentulism (one or more teeth missing in the dental arch), patients with all types of removable partial dentures, and ongoing orthodontic treatment. Patients with a history of surgical interventions, fractures, or other injuries in the area of the TMJ or jaws were also excluded from this study. None of the patients were being treated with bruxism splints at the time they were included into the study or had previous treatment with occlusal splints within the past 2 years.

### Sample size calculation and randomization

The sample size calculation (PS Power and Sample Size Calculation Program, version 3.1.6) was determined on the basis of a previous study comparing the success of TMD after different treatment options^43^. The calculation led to a required sample size of 18 patients per group to detect a significant difference (80% power, α-level 0.05)^44^. Therefore, the sample size for this study was increased to 20 patients per group to account for possible losses, making up a total of 40 patients. The patients were randomly assigned to MMR registration with the Aqualizer (AQU) or chin point guidance (CPG) using a computer-generated randomization list with a random block size of 4. The dental technician and the examiner in the recall visits were blinded to the type of MMR registration method.

### Interventions

All examinations and interventions were performed by one experienced dentist. At baseline, the presence of the following clinical TMD symptoms was assessed according to the Diagnostic Criteria for Temporomandibular Disorders (DC/TMD) for Clinical and Research Applications^4^: #1 lateral mandible deviation on opening, #2 limited jaw opening, #3 TMJ sounds, #4 TMJ pain with maximum opening, #5 pain with TMJ palpation, and #6 pain with muscle palpation (M. temporalis, M. masseter, M. sternocleidomastoideus and M. trapezius). An additive, binary scoring system (presence = 1, absence = 0) was used to create a total TMD symptom score (TSS) for each patient at each visit (baseline, 3 month follow-up, 6 month follow-up)^45^. Additionally, subjective TMD-related pain intensity was assessed by having the patients complete a numerical rating scale (NRS) ranging from 0 (“no pain”) to 10 (“worst pain imaginable”). Furthermore, the patients’ perception of the impact of the TMD-related symptoms on their quality of life (QoL) was assessed using a four-point Likert scale. For statistical analyses, a numerical value was assigned to each response option (“not at all”: 1, “to a little extent”: 2, “to a large extent”: 3 and “to a very large extent”: 4)^46^.

#### Maxillomandibular relationship registration

##### Aqualizer group (AQU)

The Aqualizer (Aqualizer Splint Systems by Jumar Corporation, Bainbridge Island, WA, USA) was placed between the upper lip and the oral vestibule of the maxilla and the patient was told to gently bite on the fluid pads for ten minutes. Once the patient asserted to have found the most comfortable position of the mandible, an anterior jig was placed over the middle upper and lower incisors (GC Bite Compound, GC Corporation, Tokyo, Japan). The Aqualizer was removed with the anterior jig still in place and bite registration material was applied to the posterior teeth (Regisil, Dentsply Sirona, Charlotte, NC, USA) to capture the designated position of the mandible.

##### Chin point guidance group (CPG)

Two cotton rolls were placed over the occlusal surfaces of both mandibular first molars and the patient was instructed to bite down for ten minutes, thus preventing the teeth from occluding. CR was recorded using the three-fingered chin point guidance technique with an anterior jig. In brief, the dentist creates a tripod with the thumb, index and middle finger at the chin symphysis and the inferior borders of the mandible and guides the patient’s mandible into a thermoplastic impression material (GC Bite Compound) placed over the maxillary central incisors by applying gentle pressure to the chin area with the thumb. After hardening of the jig, reproducibility of the designated CR position was tested by repeating the closure movement around the hinge axis several times and assuring that the lower incisors met the indentations of the jig upon each guided closure. The CR position was then secured with a bite registration material (Regisil) as described above.

#### Facebow registration and fabrication of the occlusal splints

A digital facebow (Zebris JMAnalyser+, Zebris Medical GmbH, Isny, Germany) was used to capture the patients’ axis relations and the trajectories of the temporomandibular joint (TMJ) in accordance with Posselt’s envelope of motion. Additionally, condylar guidance inclination, lateral excursion and the Bennett angle were recorded. The measurements were saved as XML data and later used for the virtual planning of the occlusal splint. Dental impressions of both jaws were taken with a polyether impression material (Impregum) to fabricate dental plaster casts. The maxillary cast was mounted in an articulator (Artex CR, Amman Girrbach AG, Koblach, Austria) according to the individual axis relations obtained with the digital facebow. The mandibular cast was mounted in the MMR position captured with the bite registration. The dental casts were digitized in the designated MMR position by a laboratory scanner (Tizian Smart-Scan Plus, Schütz Dental GmbH, Rosbach, Germany) and the occlusal splint was virtually designed with a CAD software (exocad, exocad GmbH, Darmstadt, Germany) by a dental technician, who was blinded to the type of MMR registration method. The virtual splint was exported as a standard tessellation language (stl) file and sent to a dental milling unit (Tizian Cut 5.2 plus, Schütz Dental GmbH), where the occlusal splint was milled from a PMMA blank (Astron Clearsplint, Schütz Dental GmbH) with thermoplastic properties. Finishing of the splints were performed by a dental technician. The splint was inserted into the patient’s oral cavity in a second appointment, where the accuracy of the internal and occlusal fit was examined. If any further occlusal interferences appeared, the splint was adjusted and the extent of the necessary adjustments was documented (“none”: 0, “very few”: 1, “a few”: 2, “many”: 3, “very many”: 4). All patients were instructed to wear the occlusal splints regularly at night (sleep splint).

#### Follow-up

Recall visits were scheduled after 3 and 6 months of sleep splint therapy. The same variables were assessed as at baseline: TMD symptoms #1–6 (TSS), subjective pain intensity (NRS) and the impact of the occlusal splint therapy on the patient’s QoL. The clinical examinations at the recall visits were performed by a second experienced dentist, who was blinded to the type of MMR registration method. For all palpation procedures, inter-examiner reliability was achieved by calibrating finger pressure with a force-measuring device (AFP 3100L, AE Adam GmbH, Felde, Germany) according to a previously described protocol^47^. If necessary, any occlusal adjustments were performed by the recall dentist and documented (yes/no). In addition to these parameters, all patients finishing the treatment were asked to complete a patient satisfaction questionnaire (PSQ) at the 6-month recall visit (Supplementary File 1). The response to all questions was based on a Likert scale with four possible answers (“strongly disagree”, “disagree”, “agree” and “strongly agree”).

### Outcomes

The primary outcome of this study was the alleviation of the TMD symptoms after treatment with the AQU or CPG-based occlusal splints. The variables used for judging symptom alleviation were the TSS (sum of TMD symptoms #1–6) and subjective pain intensity (NRS).

Secondary outcome variables were the patients’ perception of improvement of their QoL, patient satisfaction with the treatment and the accuracy of the occlusal fit. The occlusal fit was judged by the extent of occlusal adjustments needed upon insertion of the splint and at follow-up, and patient satisfaction was measured by evaluating the PSQ at the 6-month recall visit.

### Statistical methods

All inferential and descriptive statistical analyses were performed in Python 3.8.0 using the packages *scipy*, *scikit*, *matplotlib and seaborn*^48^. Data were tested for normal distribution with the Shapiro–Wilk test and homogeneity of variances was assessed with the Levene’s test. Mann–Whitney *U* test and Fisher’s exact test were used to demonstrate that there were no significant differences between the study populations in the AQU and CPG for any of the demographic data. For comparing changes in the average TSS and QoL within each study group, multiple group comparisons were performed with the Friedman test, followed by Conover’s post hoc test with a Holm–Bonferroni correction. Pairwise comparisons of the TSS, the NRS pain intensity and QoL between the AQU and CPG group at each visit were assessed with a Student’s *t*-test or a Mann–Whitney-*U* test where applicable. For demonstrating the magnitude of the TSS and NRS pain intensity reduction, effect sizes were estimated for each parameter by computation of Cohen’s d with a 95% confidence interval and interpreted according to the commonly used guidelines^49,50^. The alpha level was set to 0.05 for all statistical analyses.

### Ethics approval

All procedures performed in studies involving human participants were in accordance with the ethical standards of the institutional and/or national research committee and with the 1964 Helsinki Declaration and its later amendments or comparable ethical standards. This study adhered to the Consolidated Standards of Reporting Trials (CONSORT 2010) statement. The study was approved by the Ethics Committee of the Medical Faculty, University of Munich (Registration No. 20-681, 28/08/2020).

### Informed consent

Informed consent was obtained from all individual participants included in the study.

### Supplementary Information

Supplementary Information.

## Data Availability

The data that support the findings of this study are not openly available due to reasons of sensitivity and are available from the corresponding author upon reasonable request.
