# Supplementary material for: Recording the maxillomandibular relationship with the Aqualizer system prior to occlusal splint therapy for treating temporomandibular disorders: a randomized controlled trial
Source: Sci Rep. 2023 Dec 18;13:22535. doi: 10.1038/s41598-023-49911-7 (PMC10728157; doi:10.1038/s41598-023-49911-7)
Supplement: Supplementary file 1 — Supplementary Information. [file 41598_2023_49911_MOESM1_ESM.docx]

**SUPPLEMENTARY MATERIAL 1**

**Centric relation registration with the Aqualizer® system prior to occlusal splint therapy for treating temporomandibular disorders: a randomized controlled trial**

Journal: **Scientific Reports**

Karin Christine **HUTH**^a^

Alexandra **BEX**^a^

*Maximilian **KOLLMUSS**^a^

Sabina Noreen **WUERSCHING**^a^

^a^ Department of Conservative Dentistry and Periodontology, LMU University Hospital, LMU Munich, Goethestrasse 70, 80336 Munich, Germany

***Corresponding author**

Maximilian **KOLLMUSS**, PD Dr. med. dent, DDS

Department of Conservative Dentistry and Periodontology

LMU University Hospital, LMU Munich

Goethestrasse 70, 80336 Munich, Germany

Telephone: +49 89 4400 50377

e-mail address: maximilian.kollmuss@med.uni-muenchen.de

**Patient satisfaction questionnaire (PSQ)**

**How strongly do you agree or disagree with the following statements?**

Qn. 1 General satisfaction: I am satisfied with the entire treatment I received from start to finish.

| - strongly disagree | - disagree | - agree | - strongly agree |
| --- | --- | --- | --- |

Qn. 2 Expectations: My expectations to the treatment were fulfilled.

| - strongly disagree | - disagree | - agree | - strongly agree |
| --- | --- | --- | --- |

Qn. 3 Alleviation: The treatment helped alleviate my initial discomforts.

| - strongly disagree | - disagree | - agree | - strongly agree |
| --- | --- | --- | --- |

Qn. 4 Wearing comfort: I did not encounter any problems while wearing the centric relation splint.

| - strongly disagree | - disagree | - agree | - strongly agree |
| --- | --- | --- | --- |

Qn. 5 Recommendation: I recommend this type of treatment.

| - strongly disagree | - disagree | - agree | - strongly agree |
| --- | --- | --- | --- |

Qn. 6 Comfort: Centric relation registration with the Aqualizer system was not uncomfortable (please answer only if you received centric relation registration with the Aqualizer).

| - strongly disagree | - disagree | - agree | - strongly agree |
| --- | --- | --- | --- |

*PSQ translated from German*
